# Variations on the theme: focus on cerebellum and emotional processing

**DOI:** 10.3389/fnsys.2023.1185752

**Published:** 2023-05-10

**Authors:** Camilla Ciapponi, Yuhe Li, Dianela A. Osorio Becerra, Dimitri Rodarie, Claudia Casellato, Lisa Mapelli, Egidio D’Angelo

**Affiliations:** ^1^Department of Brain and Behavioral Sciences, University of Pavia, Pavia, Italy; ^2^Centro Ricerche Enrico Fermi, Rome, Italy; ^3^Brain Connectivity Center, IRCCS Mondino Foundation, Pavia, Italy

**Keywords:** cerebellum, emotion, Purkinje cell, microcircuit functioning, modular organization

## Abstract

The cerebellum operates exploiting a complex modular organization and a unified computational algorithm adapted to different behavioral contexts. Recent observations suggest that the cerebellum is involved not just in motor but also in emotional and cognitive processing. It is therefore critical to identify the specific regional connectivity and microcircuit properties of the emotional cerebellum. Recent studies are highlighting the differential regional localization of genes, molecules, and synaptic mechanisms and microcircuit wiring. However, the impact of these regional differences is not fully understood and will require experimental investigation and computational modeling. This review focuses on the cellular and circuit underpinnings of the cerebellar role in emotion. And since emotion involves an integration of cognitive, somatomotor, and autonomic activity, we elaborate on the tradeoff between segregation and distribution of these three main functions in the cerebellum.

## Emotion and the cerebellum

The term *emotion* is used to designate a collection of neurophysiological responses triggered from parts of the brain to the body and to other parts of the brain, elicited by stimuli from the external world. A collection of such responses results in an *emotional state*, defined by changes within the body. The term *feeling* is used to describe the complex mental state that results from the emotional state and reflects the ability to subjectively experience the mental states resulting from the experience of emotions (Damasio, 1998). Several studies are striving to understand the neurophysiological underpinnings of emotional processing, intended as the ensemble of mechanisms that allow emotion to be generated, learned, and managed.

There is consensus that emotion in mammals involves complex brain networks centered on structures classically referred to as the limbic system (Papez, 1937; Maclean, 1990; Nakano, 1998). These include a core composed by the cingulate cortex, amygdala, hypothalamus, and hippocampus and have been defined quite circularly by their involvement in emotion (Damasio, 1998). It has recently been noted that a discrete system of components may not be sufficient to describe emotion (Roxo et al., 2011; Barrett and Satpute, 2019; Steffen et al., 2022) and indeed the classical networks of the limbic system have been extended to include the periaqueductal gray (PAG), striatum, prefrontal cortex and parietal cortex, and cerebellum (Apps and Strata, 2015; Adamaszek et al., 2017; Rolls, 2019). The cerebellum is typically recognized for its role in movement coordination and motor learning, but increasing evidence suggests it may also be involved in higher-order functions, including emotional and cognitive processing (Schmahmann, 2004). Over the past three decades, insights into the role of the cerebellum in emotion have increased to include perception, recognition, forwarding, encoding, and learning of emotional information. These processes are thought to substantially contribute to the generation of experiences and the regulation of emotional states in relation to motor, cognitive, and social behaviors, with implications for pain, speech, and mood disorders.

In humans, neuroimaging indicates cerebellar activation in fear learning paradigms (Lange et al., 2015; Ernst et al., 2019). Structural and functional abnormalities also appear to be correlated with impaired mood regulation and anxiety disorders, generating what is known as the cerebellar cognitive-affective syndrome (here, “affective” corresponds to “emotional”) (Hoche et al., 2018). While investigations in humans are providing a privileged point of view on emotion, investigations in experimental animals are revealing most of what we know about the underlying neuronal and circuit mechanisms. Therefore, we will consider the two aspects in turn.

This review takes a physiological perspective evaluating the cellular and circuit underpinnings of the cerebellar role in emotion. And since emotion involves an integration of cognitive, somatomotor, and autonomic activity (Itô, 1984; Ito, 1993, 2005, 2008; Schmahmann, 2004; Schmahmann and Caplan, 2006; D‘Angelo and Casali, 2013; Apps et al., 2018), we will elaborate on the possible distinction, convergence, and overlap of these three main functions in the cerebellum.

## How could the cerebellum support emotional processing?

This chapter provides a succinct summary of cerebellar structure, function, and dynamics, to understand how they contribute to processing different aspects of behavior, and in particular emotion.

### Structure

**FIGURE 1:**
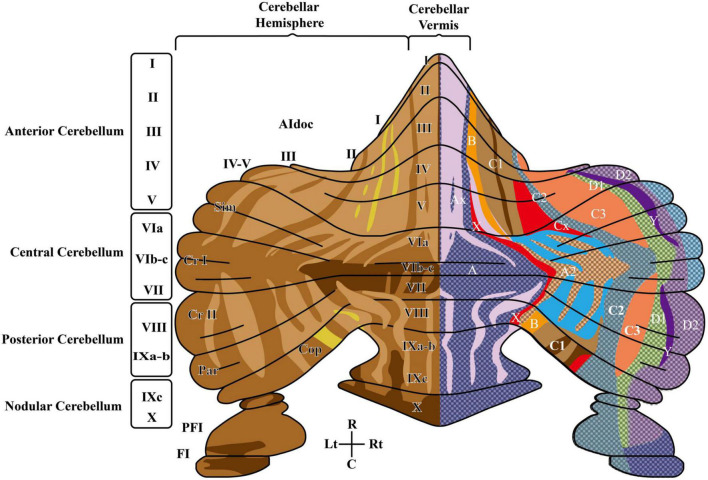
The rodent cerebellum includes the midline vermis and the lateral hemispheres. The **left side** shows the anatomical subdivisions and the striped pattern deriving from Aldoc (zebrin II) expression levels in PC (increasing expression levels from light to dark brown). In the vermis, subdivisions in the anterior-posterior orientation are delimited by circumvolutions and fissures and are further segmented into 10 lobules: the anterior cerebellum (lobules I – V), the central cerebellum (lobules VI – VII), the posterior cerebellum (VIII-IXb), nodular cerebellum (lobule IXc-X), flocculus (FI) and paraflocculus (PFI). Lobules VI-VIII expand into large hemispheric lobules including crus I and crus II. The **right side** shows the cerebellar olivo-cortico-nuclear modules defined by IO-CF-PC-CN closed-loop connectivity, identifying seven parallel longitudinal zones (A, B, C1, C2, C3, D1, D2). C, caudal; Cop, copula pyramidis; Cr I, crus I of ansiform lobule; Cr II, crus II of ansiform lobule; PFl, paraflocculus; Fl, flocculus; L, lateral; Lt, left; Par, paramedian lobule; R, rostral; Rt, right; Sim, simple lobule. Modified from Zhou et al. (2014) and Voogd (2014).

The cerebellum contains a well-defined set of cells and fibers and is organized in *parasagittal modules* and *transverse zones*, that are further subdivided based on biochemical mapping (Figure 1). The intersection of these sagittal and transverse maps generates smaller units called *cortical microzones* which, once connected to the deep cerebellar nuclei (DCN) and inferior olive (IO) neurons (see abbreviation list), form the *olivo-cortico-nuclear microcomplexes* (Apps and Hawkes, 2009; Apps et al., 2018).

**FIGURE 2:**
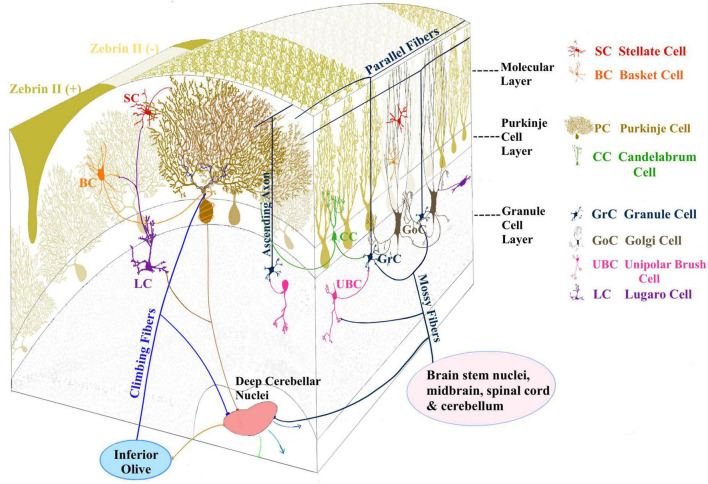
Three layers compose the cerebellar cortex: the molecular layer, Purkinje cell layer, and the granular layer. The principal inputs to the cerebellar cortex are conveyed by mossy fibers (MF) originating from different brain stem and spinal cord nuclei and climbing fibers (CF) originating from the inferior olive. MFs contact GrCs and GoCs in the granular layer. GrC axon ascends to the molecular layer and bifurcates originating the parallel fibers (PF). GrC axons contact Purkinje cells (PCs) and molecular layer interneurons (MLI). PCs provide the sole output of the cerebellar cortex and feature a wide, flat, highly branching dendritic tree and a single long axon projecting to the deep cerebellar nuclei (DCN), from where cerebellar efferent projections are sent to other brain areas. The MLI are Stellate and basket cells (SC, BC) located in the molecular layer. The candelabrum cells (CC) (Lainé and Axelrad, 1994; Osorno and Regehr, 2022) are present in the PC layer, while unipolar brush cells (UBC) and lugaro cells (LC) are reported in the granular layer.

The concept of microcomplex is rooted in the specific organization of the climbing fibers (CFs) emitted by the IO and in their projections to Purkinje cells (PCs) and DCN cells (Apps and Hawkes, 2009; Figure 2). Indeed, CFs first contact DCN cells and then branch on the sagittal plane contacting several PCs (about 7 in rodents). These PCs in turn project back to the same DCN cells receiving a common IO input and then these DCN cells, through inhibitory interneurons, project to the same IO neurons that generated the CF, forming a closed loop circuit. Mossy fiber (MFs) are the other major inputs to these circuits. Opposite to CFs, MFs spread on the transverse plane (some branches can cross the midline reaching the contralateral cerebellum) and do not respect the borders of microcomplexes. And parallel fibers (PFs), the axon of granule cells (GrCs), bifurcate in the molecular layer traveling transversally and connecting regions several millimeters away. In addition, intrinsic biochemical properties of PCs generate maps, often referred to as stripes (Apps and Hawkes, 2009), oriented along the longitudinal axis, increasing the possibility for MFs to intercept regions with different PC properties. Recently, *Zebrin II* (Aldolase C) positive and negative stripes have been identified (Figure 1). The zebrin stripes largely overlap with longitudinal cerebellar modules to generate microzones (Apps and Hawkes, 2009) and PCs within a microzone show synchronous firing when activated by the IO loops through the CFs (Witter and De Zeeuw, 2015). Functionally, zebrin stripes show *upbound* and *downbound* properties depending on CF connectivity, PC discharge regulation and PF-PC long-term depression (see below).

Thus, we have an intricate mesoscopic organization that overlaps with a broad mapping of anatomical subdivisions to bodily and mental functions. This brings about two main consequences. First, specific behavioral functions impinge on complex anatomic-functional maps made of many microcomplexes and patches rather than on simply maps residing in uniform cerebellar regions and differentiated by their input-output connectivity. Secondly, mapping of bodily and mental functions involves multiple cerebellar regions, mostly due to the distribution of MF terminal branches and PFs. These considerations are challenging the historical view that the cerebellum is made of multiple parallel non-communicating processing units, leaving space to what we may call “multimodal fusion”. As a consequence, the focus moves from the definition of cerebellar functions on a pure anatomical basis to that of the cerebellar algorithm that operates with different inputs and outputs (D‘Angelo and Casali, 2013). This algorithm, in turn, might be tuned depending on the local properties of the microcomplexes that are involved.

### Function

Independent from the nature of inputs, being them motor, sensory, cognitive, or emotional, the cerebellar algorithm *learns to predict the precise timing of correlated events* (D‘Angelo and Casali, 2013). This algorithm establishes a causal relationship between afferent signals in sequence and can apply to different contexts, e.g., correlating elementary components of movement in motor coordination, sensory stimuli in eye-blink classical conditioning or in fear conditioning, or abstract items in logical or emotional operations. In the realm of voluntary functions, the cerebellum operates in a similar way by comparing *intention* (conveyed through descending cortico-cerebellar pathways) with *execution* (conveyed by afferent sensory fibers) to predict possible errors. This *de facto* anticipates and corrects execution errors, generating the precise predictive motor control typical of vertebrates. These observations, combined with insightful theories, have been extended to explain fluid reasoning and mental coordination and are arguably important also for emotional control.

### Dynamics and mechanisms

On the mechanistic side, the main issue is how the cerebellar units process input signals through their internal algorithm. In other words, how do cerebellar neurons and synapses implement such algorithm. This issue has been addressed by several physiological and computational works, revealing a complex set of neuronal and synaptic properties that can generate the input-output transformation and plastic changes required to process the input signals (for review see De Schepper et al., 2022). The circuit operates a complex spatio-temporal transformation of the MF input and uses the CF input to drive plasticity. On top of this, the circuit governs intrinsic oscillatory dynamics that allow the time correlation of the processes involved (D’Angelo, 2011; D’Angelo et al., 2011).

### A unified framework for motor, cognitive, and emotional processing?

The intricate neuronal circuitry of the cerebellum is thought to encode internal models that reproduce the dynamic properties of body parts allowing the brain to precisely control the movement without the need for sensory feedback. It is thought that the cerebellum might also encode internal models that reproduce the essential properties of mental representations elaborated in the cerebral cortex. This hypothesis suggests a possible mechanism by which intuition and implicit thought might function and explains aspects of cognitive and emotional reasoning as well as symptoms exhibited by psychiatric patients (Ito, 2008).

Recently it was argued that a similar circuit structure in all cerebellar areas may carry out various operations using a common computational scheme (D‘Angelo and Casali, 2013). The authors attributed different roles of the cerebellum to the specific connectivity of the cerebellar modules with motor, cognitive, and emotional functions at least partially segregated into different cerebro-cerebellar loops. This led to formulate a *meta-levels hypothesis*: from cellular/molecular to network mechanisms leading to generation of computational primitives, thence to high-level cognitive/emotional processing, and finally to the sphere of mental function and dysfunction. It has been proposed that the intimate interplay between timing and learning (reminiscent of the “timing and learning machine” capabilities long attributed to the cerebellum) reverberates from cellular to circuit mechanisms. Subsequently, integration within large-scale brain loops could generate the disparate cognitive/emotional and mental functions in which the cerebellum is implicated. The cerebellum should therefore operate as a general-purpose co-processor, whose effects depend on the specific brain centers to which individual modules are connected. Abnormal functioning in these loops could eventually contribute to the pathogenesis of major brain pathologies including not just ataxia but also dyslexia, autism, schizophrenia, and depression. These functions and dysfunctions are deeply involving cognitive and emotional processing.

### A system view

In engineering, the cerebellum has been assimilated to a double *forward and inverse controller*, capable of predicting system states based on contextual information and previous memory (Ito, 1993, 2008). The fact that our understanding of cellular phenomena is sufficient to explain the predictive capabilities of the system is demonstrated by the ability of neuro-robots, embedding a canonical cellular representation of the cerebellar circuit dynamics and mechanisms, to reproduce a wide set of sensory-motor control tasks (Casellato et al., 2015; Antonietti et al., 2016, 2018, 2022). This same system approach may be applied to cognitive and emotional processing, provided the controller, to which the cerebellar circuit is connected, is appropriately designed and implemented. Eventually, a precise representation of the neuroanatomical principles based on cerebellar connectivity and subdivisions, may help understanding the computational principles and mechanisms underlying the cerebellar involvement in emotion (D’Angelo et al., 2016; D’Angelo and Jirsa, 2022).

## Cellular and circuit variants in the rodent cerebellum

Since the cerebellar computational algorithm is likely to be tuned depending on the local properties of microcomplexes, it is important to consider how circuit properties vary across cerebellar regions. This section is based on rodent studies, in which accurate investigations have addressed the molecular and cellular mechanisms and the potential sources of heterogeneity underlying functional specialization.

Anatomical and physiological studies have shown that the midline cerebellar vermis is particularly involved in emotional processing and that lesions and other interventions on this cerebellar compartment affect emotion-related behaviors (reviewed in Apps et al., 2018). The cerebellar vermis receives olivo-cerebellar (CF) input from the caudal medial accessory olive and emits a cortico-nuclear output to the medial (fastigial) cerebellar nucleus which, in turn, has widespread projections to the cingulate cortex, prefrontal cortex, parietal cortex, amygdala, hypothalamus, hippocampus, periaqueductal gray (PAG), and striatum (Voogd and Ruigrok, 2004; Blatt et al., 2013; Rolls, 2019) (see also next chapter).

Different cerebellar vermal lobules are associated with different aspects of emotion (see Figure 1). From rostral to caudal: lobules IV–VI with fear learning/memory and affective state; lobule VI–VII with orientation of gaze; lobule VIII with fear-induced freezing behavior; and lobule IX and X with cardiorespiratory control (Apps et al., 2018). Different parts of the vermis could therefore regulate and integrate the cognitive, motor, and autonomic aspects of fear-related behavior (Clausi et al., 2017).

Here, neuron and microcircuit variants are considered in turn and a summary of properties that may be more relevant to emotional processing is reported in the section on “Conclusion”.

### The cerebellar microcircuit and its variants

**FIGURE 3:**
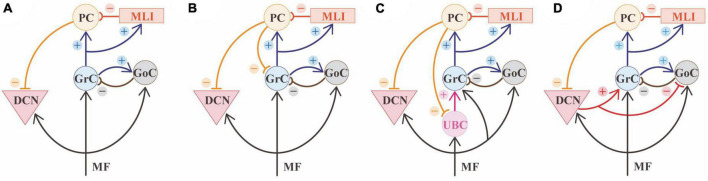
Cerebellar microcircuit variants. **(A)** Canonical circuit of the cerebellum. The primary input of mossy fibers (MF) project to deep cerebellar nuclei (DCN), granule cells (GrC), and Golgi cells (GoC). GoC inhibits GrC in a feedforward and feedback loop. Besides GoC, GrC axons activates Purkinje cell (PC) and molecular layer interneurons (MLI). PCs provide the sole output of the cerebellar cortex and inhibit the cerebellar nuclei. **(B)** A first variant of the canonical circuit is represented by PCs axon collaterals projecting to GrC in the granular layer. **(C)** A second variant is represented by unipolar brush cells (UBC), that are contacted by MF and excite GrC. UBC receive inhibition through PC axon collaterals. **(D)** A third variant shows the nucleo-cortical projections: DCN axon collaterals contact GrCs with excitatory connections and inhibit GoCs, in the granular layer.

The cerebellar circuit includes several cell types connected according to the general circuit scheme shown in Figures 2, 3A. A recent modeling work summarized the general knowledge on the cerebellar microcircuit generating a reference ground-truth that binds together structure, function, and dynamics (De Schepper et al., 2022). In the granular layer, each glomerulus hosts ∼50 excitatory and ∼50 inhibitory synapses on as many GrC dendrites, plus ∼2 excitatory synapse on basolateral dendrites of as many Golgi cells (GoCs). Each one of the 4 GrC dendrites receives an excitatory and (in most cases) an inhibitory input from as many different MFs and GoCs, respectively. Each GoC receives ∼320 ascending axon (AA) synapses on basolateral dendrites and ∼910 PF synapses on apical dendrites, and there are ∼7-8 electrical synapses plus 160 GABAergic (using the Gamma-aminobutyric acid neurotransmitter) synapses per GoC-GoC pair. In the molecular layer, ∼25% of AAs contact the distal dendrites of the overlaying PCs (each AA forming 2.4 synapses on average), while PFs form 1 synapse per PC dendritic intersection. In summary, each PC receives 12% of the whole GrC inputs from AAs. Among the molecular layer interneurons (MLI), we consider the Stellate cells (SCs) and basket cells (BCs) populations. There are ∼25 SC-PC and BC-PC synapses altogether. Moreover, there are $∼17\dot{6}00$ PF-MLI-PC synapses ($∼2\dot{6}00$ PF-SC-PC and $∼15\dot{0}00$ PF-BC-PC synapses).

Beyond the canonical description of the cerebellar network reported above (Figure 3A), there is growing evidence that the cerebellar cortex is not homogenous in structure, physiology, or gene expression (Nishiyama and Linden, 2004; Ito, 2006; Sillitoe et al., 2009; Fujita et al., 2012; Martinez et al., 2013; Nedelescu and Abdelhack, 2013; Chen et al., 2022) and these variations of regional features (Figure 3) may influence information processing. Several cell clusters have recently been reported based on transcriptomic profile: there are 9 PCs clusters overlapping with zebrin stripes, 3 GrC clusters showing antero-posterior segregation (anterior-central zone, central-posterior zone, nodular zone), 2 GoC clusters, and two clusters of MLI including both SCs and BCs (Kozareva et al., 2021).

#### Purkinje cells

Two large groups of PCs were identified based on the expression of Aldoc (aldolase C, the enzyme responsible for the zebrin II pattern) (Figure 3). The Aldoc positive and negative PCs define the “zebrin stripes”, consisting of longitudinal alternating bands of positive (Z +) and negative (Z-) PCs (see Figure 1). This distribution is typical of the anterior and posterior cerebellar zones (comprising lobules I - V, and VIII - dorsal IX, respectively), while the central and nodular zones (lobules VI - VII and ventral IX - X) are uniformly characterized by Z + PCs. From the functional point of view, Z- PCs are characterized by higher spontaneous firing compared to Z + PCs (Xiao et al., 2014; Zhou et al., 2014). More recently, Z- PCs were shown to undergo simple spike suppression (*downbound*) (Wu et al., 2019) and Z + PCs to undergo simple spike facilitation (*upbound*) (De Zeeuw, 2021).

In addition to heterogeneity between stripes, PCs also display functional variations along the transverse axis. PCs in the anterior lobes have more pronounced adaption of tonic firing compared to those in the nodular zone (Kim et al., 2013), where PCs show lower firing rates and reduced excitability (Witter and De Zeeuw, 2015). These differences likely make PCs in the nodular lobe more suitable to integrate vestibular inputs, which are slower compared to the faster and stronger inputs arriving in the anterior zone. An anteroposterior gradient has been also reported for the regularity of firing (increasing toward the nodular zones) (Witter and De Zeeuw, 2015). The difference in PCs firing along the transverse axis may simply match the different distribution pattern of zebrin, with more zebrin negative PCs in the anterior cerebellum, and more positive PCs in the posterior and nodular cerebellum.

PCs regional diversity also impacts the microcircuit connectivity. In addition to the general scheme, in which granule cells activate PCs and DCN cells in sequence, recent evidence showed that PCs axon collaterals in the granular layer form functionally active contacts onto GrCs (Guo et al., 2016) and Unipolar Brushed Cells (UBCs) (Guo et al., 2021) that are less represented in the anterior cerebellum (Figures 3B, C). Moreover, these collaterals show more complex morphology and more numerous contacts in the granular layer starting from lobule VI toward lobule X (Guo et al., 2016). This connection is characterized by a low probability of GABA release, suggesting that a robust activation of PCs is needed to activate this additional source of feedback inhibition to GrCs. These results show that, in lobules VI-X, PCs contribute to both phasic and tonic inhibition in the granular layer, therefore affecting the spatiotemporal dynamics of GrCs firing (D‘Angelo et al., 2013; Mapelli et al., 2014).

#### Unipolar brush cells

The canonical cerebellar microcircuit also differs based on the regional distribution of UBCs (Figure 3C). UBCs are excitatory neurons involved in the MF-GrC pathway. These neurons are located in the granular layer, with highest density in the medial cerebellum, particularly lobules I, IX, X, and the lowest density in the lobules IV-VI in mammals (Takács et al., 1999; Mugnaini et al., 2011). UBCs receive a robust synaptic input by a single MF and contact several GrCs, and are therefore called “intrinsic mossy fibers” (Nunzi and Mugnaini, 2000). Their response patterns amplify the vestibular inputs, significantly modifying the transmission pathway from MFs to PCs (Nunzi and Mugnaini, 2000; Locatelli et al., 2013; Balmer and Trussell, 2019).

#### Granule cells

GrCs functions and connectivity are heterogeneously distributed in different subregions of the cerebellum. First, GrCs receive excitatory inputs from UBCs, where these neurons are enriched (mainly in the nodular zone). Second, PCs axon collaterals contribute to GrC inhibition with a regional gradient increasing toward posterior and nodular zones. It has also been reported an antero-posterior gradient for GrCs efficiency in responding to burst vs. tonic inputs, based on the differential expression of the CaV3-Kv4 complex (Heath et al., 2014; Witter and De Zeeuw, 2015). This finding probably matches the differences in the inputs (with slower vestibular inputs arriving at the posterior and nodular zones). Moreover, GrCs receive excitation from nucleo-cortical projections in several lobules, with a sagittal arrangement (Houck and Person, 2014), which generates an internal amplification loop (Gao et al., 2016).

#### Golgi cells (and molecular layer interneurons)

Cerebellar inhibitory interneurons also show some specific spatial arrangement, in particular concerning GoCs. GoC dendrites are topographically organized within striped boundaries determined by the zebrin II expression patterns in PCs. There is no evidence in literature that different GoC subtypes are present in positive and negative stripes, though two clusters have been identified based on the transcriptomic profile (Kozareva et al., 2021). Moreover, a recent study highlighted the functional heterogeneity of GoCs depending on the expression of the human glycine neuronal transporter type 2 (GlyT2), affecting the inhibitory control over GrCs (Dumontier et al., 2023). Additionally, GoCs receive inhibition from nucleo-cortical projections in several lobules (Ankri et al., 2015), decreasing GoC inhibition over GrCs. MLIs are involved in complex inhibitory loops that shape PC responses (Brown et al., 2019; Prestori et al., 2019). To the best of our knowledge, a differential spatial distribution of MLIs subtypes in different zones or lobules has not been reported, yet.

#### Deep cerebellar nuclei cells

The cellular heterogeneity in the DCN is less studied, compared to the cerebellar cortex. Nevertheless, DCN can be subdivided based on the connectivity pattern in caudo-ventral and rostro-dorsal axes, based on innervation from zebrin II positive and negative PC, respectively [for a comprehensive review on this subject see Kebschull et al. (2023)]. Different functional nucleo-cortical projections have also been identified in several lobules, arranged in a sagittal organization mainly matching the territories of PCs sending inputs to those DCN regions (Houck and Person, 2014). Therefore, nucleo-cortical projections (Figure 3D) match the cortico-nuclear ones, originating closed loops, but the amount of reciprocal connections can differ in different regions (Houck and Person, 2014). Excitatory and inhibitory projections from DCN affect GrCs and GoCs activity, respectively (Ankri et al., 2015; Gao et al., 2016). Anatomical tracing studies in rats reported that certain regions of the cerebellar cortex seem to lack nucleo-cortical projections, such as the lateral vermis (longitudinal zone B, see Figure 1), the lateral paravermis (C3 zone), and some patches of the paravermis C1 zone and the hemisphere (D zone) (Buisseret-Delmas and Angaut, 1988, 1989). As a result, each lobule shows different patterns of connectivity. For example, in cats, Lobule V has been reported to lack nucleo-cortical projections in the C1 zone (medial paravermis) (Trott et al., 1998a) and be enriched in them in the C2 zone (lateral paravermis) (Trott et al., 1998b). Inside zones, the distribution can be uneven, too. For example, the C2 zone of the paraflocculus lacks nucleo-cortical projections (Trott et al., 1998b). Further anatomical and functional characterization of these projections is needed to clarify the specific impact on regional processing.

### Structural variants

In addition to differences in local microcircuit connectivity, there are also differences in the density and distribution of the different cell types as well as in the thickness of cerebellar regions.

#### Cell types and density

Geographical differences in PC pertain to neuronal locations, packing density and diameter, as well as axon and dendritic arbor morphology. In rats, there are fewer PCs at the base of each cerebellar folium than at the apex (Braitenberg and Atwood, 1958; Eccles, 1967; Armstrong and Schild, 1970), and the packing density of PCs in the anterior lobe is greater than that of the posterior lobe (Armstrong and Schild, 1970). For example, Lobule X is known to have a greater PCs density than other lobules, such as lobules II and VI (Keller et al., 2018). There are also significant regional differences in PC size, with larger PC diameter and organelle volume in phylogenetically older cerebellar regions (such as the vermis) (Parma, 1969; Müller and Heinsen, 1984). Consistent changes in PC axons diameter between white matter compartments and dendritic arbor morphology at the base of a folium compared to the apex have been described (Voogd, 2011; Nedelescu and Abdelhack, 2013; Nedelescu et al., 2018). The biophysical parameters and energy consumption of individual PC in various cortical regions could be drastically altered by these variations. Similarly, GrCs exhibit regional variations in packing density, higher in the apex than in the base of a folium, and bigger cell size in the vermis than in the hemispheres (Friede, 1955; Cerminara et al., 2015). The packing density of GoCs in a variety of mammals, including humans, is lower in the hemispheres (except for the flocculus) than in the vermis, where the densities are highest in the flocculus and lobules IX (Lange, 1974; Palay and Chan-Palay, 1982). Moreover, GoCs in the hemisphere are smaller than those in the vermis (Geurts et al., 2001, 2003).

#### Cortical thickness

On average, the granular layer is 73% thicker than the molecular layer (Zheng et al., 2022), whereas the Purkinje cell layer is single cell thick. The difference in layer thickness constrains the area occupied by neurons on the parasagittal surface, e.g., size and width of PCs dendritic trees. Clearly, synapse development and maturation as well as plasticity impact the branching and elongation of dendrites (Takeo et al., 2021). Difference in layer thickness will also impact the number of neurons, as in the granular layer.

Granular layer thickness changes from the anterior to posterior cerebellum, being much larger at lobules VI-VIII than lobules I-V and smaller in lobules IX -X than in the flocculus (Zheng et al., 2022). In the depth of the sulci, all three layers are thicker than at the crowns of the gyri. Globally, the lateral-posterior-inferior area has a thicker granular layer than the medial-superior region.

Unique PCs dendritic tree shapes in the sulcus result in more arbor field overlap between nearby PCs than at the apex (Nedelescu and Abdelhack, 2013) and cause quantitative variations in dendritic branching patterns between lobules V and IX. Two physically different PC subtypes have been identified. Type 1 PCs adhered to the conventional concept of a PC morphology, with a single primary dendrite and a rather uniform branching density across the molecular layer’s thickness. On the other hand, Type 2 PCs have two major dendrites and little branching in the bottom portion. PCs in the anterior lobe (lobule V) of adult mice exhibited a preponderance of type 1 dendritic arbors, while the posterior lobe (lobule IX) contained a bigger proportion of type 2 isoforms. Overlapping in adjacent PC arbors varies across lobules, with more overlap in lobule V than in lobule IX, and arbors in lobule V using surrounding space more efficiently than those in lobule IX. Those distinctions in neurons may throw out the potential question concerning the afferents system differentiation. For example, lobules IV and V are the key area receiving significant MF projections from the lateral reticular nucleus, while lobules VI-VIII and IX get a less dense distribution of this input (Wu et al., 1999).

### Atlas data for microcircuit and structural variations

**FIGURE 4:**
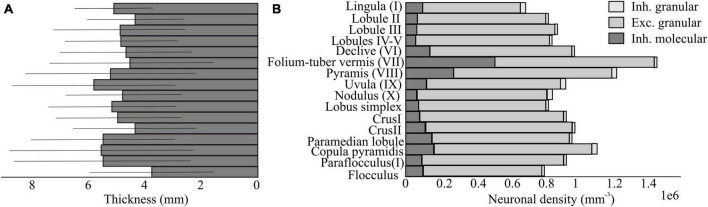
Neuron density and thickness in the mouse cerebellum based on the Blue Brain Cell Atlas. **(A)** Estimations of the thickness of the molecular layer in mm for each subregion of the cerebellar cortex, using the algorithm proposed by Rodarie et al. (2022). **(B)** Estimations of the density of excitatory and inhibitory neurons in the molecular and granular layer for each region of the cerebellar cortex. The data are based on the publicity available Blue Brain Cell Atlas (2018) website.

The cerebellum holds remarkable variations in cell morphology, density, volume, and cellular properties. These variations are further enriched considering microcircuit connectivity of different cerebellar regions, that go by lobules, stripes, modules and eventually identify specific microzones and microcomplexes. Using atlases introduce a new method to combine evidence collected through the different studies. For example, the extensive datasets from the Allen Brain Institute (ISH Data., 2023: Allen Brain Atlas: Mouse Brain.) and Blue Brain Cell Atlas (Rodarie et al., 2022) provides exhaustive information on cell types and densities that might be used for detailed model reconstructions (Figure 4). This information must now be crossed with that on cerebellar regional connectivity and behavioral localization of functions addressing those regions and pathways that are critical for emotional processing.

### Cerebellar circuits in rodent emotional behavior

**FIGURE 5:**
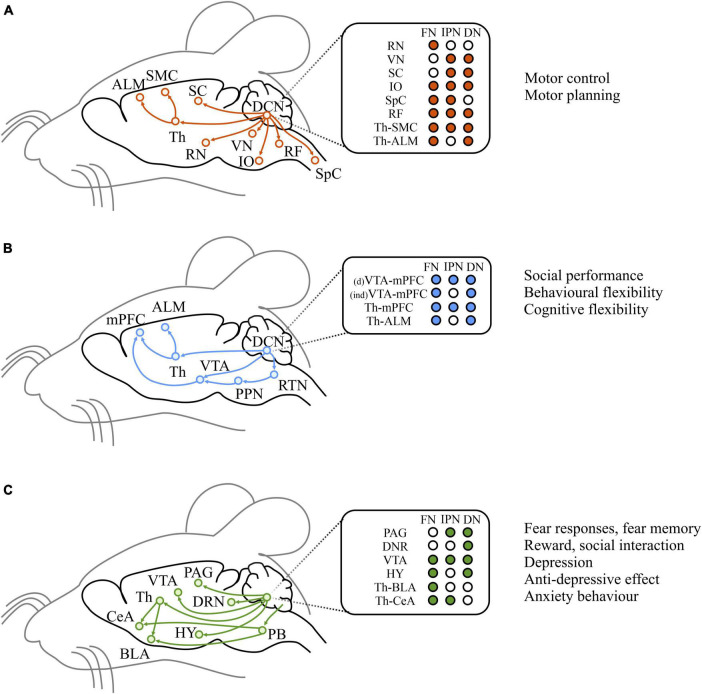
Efferent pathways from the cerebellum. Left: Schematic diagrams of historically studied efferent pathways related to motor functions **(A)**, and of direct or indirect efferent pathways that are considered to be associated with cognitive **(B)** and emotional **(C)** functions. Middle: target brain regions from each DCN. Right: summary of broad functions of the pathways illustrated on the left. ALM, anterior lateral motor cortex; SMC, sensorimotor cortex; SC, superior colliculus; Th, thalamus; RN, red nucleus; VN, vestibula nucleus; IO, inferior olive; RF, reticular formation; SpC spinal cord; mPFC, medial prefrontal cortex; VTA, ventral tegmental area; PPN, peduncolopontine tegmental nucleus; RTN, reticulotegmental nuclei; DRN, dorsal Raphe nucleus; HY, hypothalamus; PB, parabrachial nucleus; CeA, central amygdala; BLA, basolateral amygdala; PAG, periacqueductal gray. Modified from Kang et al. (2021).

While the cerebellum has canonically been implicated in motor control and motor learning, which it exerts through a set of well-studied circuits and pathways (Figure 5A), neuroimaging and clinical data recently identified the cerebellum as a key region among the emotion and cognitive relevant structures (Strick et al., 2009; Apps and Strata, 2015; Strata, 2015; Adamaszek et al., 2017). Anatomical studies in rodents have highlighted indirect connections from the DCN, the sole output of the cerebellum, to various brain structures involved in emotional (Figure 5B) and cognitive processing (Figure 5C). For example, monosynaptic projections from the DCN to the ventral tegmental area (VTA), a structure included in the dopaminergic reward system, have been described (Carta et al., 2019). Cerebellar output to the VTA mainly originates from the dentate nucleus and, to a lesser extent, from the interpositus nucleus (Baek et al., 2022). The optogenetic activation of the cerebellum-VTA pathway is rewarding and can condition mice behavior during place preference and social interaction tests (Carta et al., 2019; Baek et al., 2022). Moreover, the cerebellum and hypothalamus are highly interconnected through direct and indirect routes. The reciprocal cerebellar-hypothalamic pathway has been suggested to provide circuits through which the cerebellum can influence the autonomic hypothalamic processes activated during emotional states (Dietrichs and Haines, 1989; Gritti, 2013). In fact, the cerebellum, via its connection with the hypothalamus, is involved in controlling vasomotor reflexes and blood pressure, carotid sinus reflexes, and the somatic and autonomic manifestations of shame rage, pupil nictitating membrane, respiration, gastrointestinal functions (Zhu et al., 2011). Although a strong functional connectivity between the cerebellum and a key affective center, the amygdala, have been observed (Supple et al., 1987; Tovote et al., 2015; LeDoux, 2000), the anatomical substrate for this connectivity is still unclear. In fact, no direct connections between the cerebellum and the amygdala exist. Nevertheless, a putative di-synaptic pathway between the DCN and the basolateral amygdala (BLA) through the thalamus (mainly medial dorsal nucleus, centro-median, and parafascicular) has been described (Gornati et al., 2018). Therefore, the cerebellar output might influence the BLA, known to process affect-relevant salience and valence information (Jung et al., 2022).

### Studying cerebellar role in fear using animal models

Rodent behavioral studies have been extensively used to investigate neurobiological aspects of emotions (see Box “Emotional tasks in rodents”). However, finding valid and objective measures of animal emotions can be challenging. The most widely used emotional behavioral tests in rodents aim to trigger a fear response (Fi. In fact, fear tests elicit a clear behavioral readout and are highly reproducible. In fear conditioning, rodents show a robust conditional response during fear memory retrieval which is manifested in long periods of freezing behavior. Fear conditioning is an associative learning task. For conditioning to occur, pathways transmitting the conditioned stimulus (CS) and unconditioned stimulus (US) have to converge in the brain. It is widely believed that the amygdala is the site of CS-US convergence (LeDoux et al., 1988; Koutsikou et al., 2014). Auditory (LeDoux, 1990; LeDoux et al., 1990; Mascagni et al., 1993; Romanski and LeDoux, 1993) and other sensory inputs representing the information about the CS terminate mainly in the lateral nucleus of the amygdala (LA). LA, in turns, project to the central amygdala (CeA) which controls the expression of fear response through its projections to the brainstem. Fear conditioning to auditory CS is thought to be mediated by these pathways. In contextual fear conditioning, CS is represented by both the auditory and the context cue. Information on the contextual stimulus involves communication between the hippocampus and the basal and accessory basal nuclei of the amygdala (LeDoux, 1992, n.d.). These nuclei project to the CeA which controls the expression of the response. Somatosensory information concerning the US, including the nociceptive stimuli generated by the footshock (commonly used in fear conditioning tests), are transmitted from cortical and thalamic areas to the LA (Turner and Zimmer, 1984; McDonald, 1998), mirroring the CS pathway. Moreover, CeA receives nociceptive inputs from the parabrachial area (Bernard and Besson, 1990) and directly from the spinal cord (Burstein and Potrebic, 1993). CeA ultimately projects to different brain areas to drive the expression of fear response. Particularly, CeA modulates the activity of the bed nucleus of the stria terminalis on the release of the pituitary-adrenal stress hormone (LeDoux et al., 1988; Herman et al., 2005; Pruessner et al., 2010), and the lateral hypothalamus on the control of blood pressure. In addition, CeA activates the ventrolateral periaqueductal gray (PAG) and elicit freezing response. The PAG has long been implicated as the organizer of behavioral components of the response to threat. It exerts direct control of freezing via glutamatergic projections to premotor neurons targets in the magnocellular nucleus of the medulla. Freezing behavior can be elicited from the ventrolateral periaqueductal gray (vlPAG) in decerebrate animals, therefore descending projections are necessary and sufficient to elicit such behavioral response (Keay and Bandler, 2001). The cerebellum may also participate in the freezing response. Indeed, anatomical evidence of projections from the PAG to the cerebellar cortex have been reported (Dietrichs, 1983), as well as direct projections from the cerebellar nuclei to the vlPAG.

**BOX 1**

**Emotional tasks in rodents**

**FIGURE 6:**
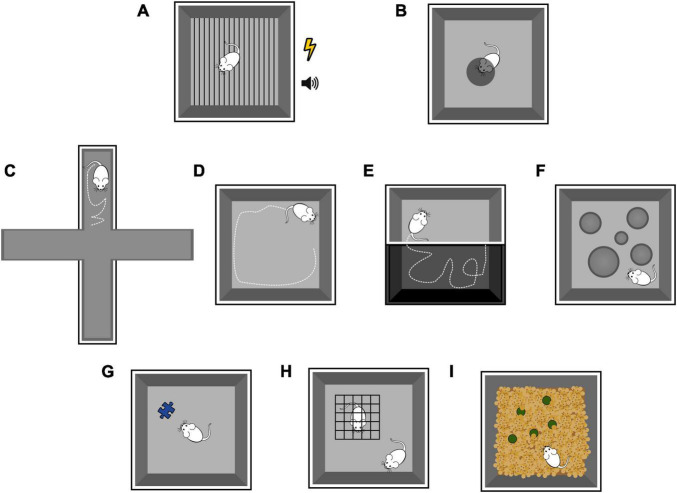
Schematic representation of emotional tasks in rodents, as detailed in the Box. Fear conditioning **(A)**. Looming test **(B)**. Elevated plus maze **(C)**. Open field test **(D)**. Dark-light box test **(E)**. Hole board test **(F)**. Novel object test **(G)**. Social novelty test **(H)**. Marble burying test **(I)**.

Fear conditioning (FC) is a type of associative learning, in which rodents learn to associate a neutral stimulus (conditioned stimulus, CS; more often a tone but sometimes a visual stimulus) with an aversive stimulus (unconditioned stimulus, US; usually a mild electrical foot shock), and show a consistent behavioral response (conditioned response) (Figure 6A). The conditioned response is the freezing behavior, defined as the complete absence of movements except those related to breathing for at least 2 seconds. The FC protocol consists first of an acquisition phase, often performed on day 1, where the animal is presented with a set of CS-US pairings and. learns to fear both the tone and the training context. FC is learned rapidly, and after one conditioning session a very stable long-lasting behavioral change is produced (consolidation). The next day, the amount of freezing during the presentation of CS alone (the tone) is used as a behavioral readout of memory retrieval. Depending on the goal of the study, the retrieval phase can be performed in the same training environment as the conditioning, with or without the auditory cues (context FC, for hippocampal-dependent learning), or in a different environment, to rule out the contribution of the context in fear memory retrieval (amygdala-dependent learning). Moreover, consolidation and stability of the fear memory can be assessed by performing the retrieval phase at some distance from the acquisition (delayed FC). Finally, fear memory can undergo extinction when CS is presented alone for enough times (extinction phase).

The looming test (Figure 6B) is a non-associative fear test that triggers rodents innate behavioral response to approaching aerial predators. The innate fear response does not require any previously acquired conditioning. A rodent is placed into an arena with a display monitor covering most of the ceiling, and an opaque/dark nest in a corner of the arena representing a hiding spot. The looming stimulus consists of a black disc appearing on the display monitor above the animal and expanding, mimicking the shadow of an approaching predator. This setting reliably and consistently triggers one of two defensive behaviors: escape toward the hiding spot or freezing for a prolonged period. The sensory modality involved in triggering the behavioral response is vision. In particular, a subpopulation of neurons in the superior colliculus seems to be necessary and sufficient to orchestrate these dimorphic defensive behaviors via two distinct pathways, eventually targeting a different nucleus in the amygdala (Wilensky et al., 2006; Tovote et al., 2015; Shang et al., 2018). Interestingly, pairing a neutral tone (CS) with a looming stimulus (US) fails to drive the associative learning of the defensive response, in contrast with the robust learned response of the classical FC (Heinemans and Moita, 2022). However, comparison between innate and learned fear studies suggest considerable overlap between circuit for innate and learned freezing and escape response, thus leaving the topic open for further investigation.

In the predator odor test, rodents are exposed to a predator odor, a natural threat, and show an increase in fear behavior, particularly freezing and avoidance. Olfaction is the most preserved sense throughout mammalian species and is the most prominent sensory modality in rodents. Moreover, early neuroanatomists and scientists recognized a strong link between olfactory processing and emotions, including the olfactory areas in the emotional circuit (Papez, 1937). Indeed, later studies confirmed the role of the connections between olfactory structures and specific nuclei in the amygdala in predator odor-induced fear behavior, including freezing. Nevertheless, the predator odor test has been progressively disregarded, possibly due to the issues in standardizing the odor stimulus, the high variability in the behavioral response, and the low reproducibility of the test.

**Anxiety tests**

Elevated plus maze, Open field test, Dark-light box test, Hole board test, Social novelty test, Novelty exploration, Marble burying test.

An array of behavioral tests has been developed to investigate anxiety-like behaviors in rodents, so called because they resemble anxiety behaviors in humans (although in rodents they might represent something else entirely). In humans, anxiety and fear produce similar behavioral responses, including but not limited to increasing vigilance, freezing and/or hypoactivity, elevated heart rate, and suppressed food consumption. As opposed to fear, anxiety is elicited by aversive stimuli or a sense of threat which are diffuse, unpredictable, and of long duration. In rodents, anxiety-like behavior is assessed by measuring the response to a novel and potentially threatening environment or object. Anxiety tests depend on locomotion and take advantage of the innate aversion rodents show for bright light and open areas. This is the case for approach-avoidant paradigms such as Elevated plus maze (Figure 6C), Open field test (Figure 6D), Dark-light box test (Figure 6E), and Hole board test (Figure 6F). These tests assess the natural exploratory behavior of the rodents, which is supposed to be reduced or suppressed when the animals feel a threat. Other widely used tests rely on the curiosity towards new objects (Figure 6G) or social novelty (Figure 6H), which is altered by stress and anxiety. More creative and less used tests, such as the Marble burying test (Figure 6I), exploit rodents’ tendency to dig the bedding of their cages and hide objects when stressed.

Often, these tests are inconclusive and non-reproducible because of the high inter-animal and inter-session variability of the results. These inconsistencies are attributed to the individual genetic variations (as genetic background and strains), testing environment (handling, testing rooms, equipment), protocols, and the rearing environment of the rodents (housing environment).

Based on early clinical and anatomical evidence, initial works were conducted on the vermis. Using inactivation procedures, the interpositus nucleus appeared to be involved in memory formation of the freezing response to acoustic CS, whereas the vermis appeared to be involved in the memorization of the freezing response both to acoustic CS and in contextual tests (Sacchetti et al., 2002). Furthermore, fear conditioning elicited long-term potentiation at PF-PC synapses, in particular at cerebellar lobules V and VI but not in IX and X. Interestingly, fear conditioning did not modify CF synapses onto PC (Sacchetti et al., 2004).

Moreover, *in vivo* field potential mapping techniques in anesthetized rats revealed potent physiological connections between vlPAG and cerebellar vermal lobule VIII (Koutsikou et al., 2014). In fact, electrical stimulation of vlPAG on one side of the brain evoked field potentials on the cerebellar cortical surface. Responses were largest in vermis lobule VIII, predominantly localized laterally in the VIII, both sides. This response is thought to be mediated by CFs activation since barbituric anesthesia, used in the study, likely weakened MFs activity. When lobule VIII was inactivated right after the animal conditioning to CS-US pairings, fear memory retrieval showed a reduction in duration of freezing behavior to the CS tone only. It has been hypothesized that the inactivation of lobule VIII during the consolidation of the fear memory resulted in the inability to retrieve the associative memory. However, a reduction in freezing duration also occurred when CS-only memory retrieval test was replaced with an innate fear test (predator odor).

### Cerebellum in autonomic response to fear

Response to fear also includes body reactions as heart rate and respiratory rate changes, vasomotor activity/blood flow modification, and consequently sweating, trembling muscles, increased body temperature (Nisimaru, 2004). The main pathway known to mediate these effects travels from the amygdala to the hypothalamus which in turns activates the pituitary gland to secrete hormones in the bloodstream and the autonomic nervous system.

In order to acknowledge the complex pattern of body reactions to fear, Signoret-Genest and colleagues recently introduced the concept of cardio-behavioral defensive state (Signoret-Genest et al., 2023). From this perspective, freezing is not considered an isolated, independent response to fear, rather the behavioral component of a complex response pattern to fear. Such complex response comprises rapid microstates associated with specific behavior and heart rate dynamics, both affected by long-lasting macrostates reflecting context-dependent threat levels. If the cerebellum has a role in freezing, which is part of such cardio-behavioral response, then it might have a role in the other bodily responses to fear. Indeed, the cerebellum has been proven able to influence autonomic functions. For example, the electrical stimulation of the vermis lobule IX causes changes in blood pressure and heart rate, together with inhibition of the baroreceptor reflex (Bradley et al., 1991).

On this matter, the direct and indirect projections from the cerebellum to the hypothalamus, spinal visceral nuclei, dorsal motor vagal nucleus, nucleus tracti solitari, parabrachial nucleus, raphe nucleus, ambiguous nucleus, are expected to take part (Zhu et al., 2011).

Furthermore, recent works have highlighted a strong functional connectivity between the cerebellum and a region of the ventro-lateral brainstem known as the pre-Botzinger complex (Lu et al., 2013). The pre-Botzinger complex is considered the central pattern generator of the respiratory rhythm (Smith et al., 1991) and possibly the site of integration and coordination of different orofacial behaviors in mice, such as whisking, licking and breathing (Lu et al., 2013; Romano et al., 2020). Moreover, pre-Botzinger complex inhibitory neurons modulate sympathetic vasomotor neuron activity, generating heart rate and blood pressure oscillations in phase with respiration (Menuet et al., 2020). Although there are no direct projections from the cerebellum to the pre-Botzinger complex (Teune et al., 2000), cerebellar nuclei project to the gigantocellular reticular formation and to the parabrachial complex, which are both downstream of the pre-Botzinger complex. In turns, these areas project to motor neurons in the spinal cord to drive orofacial movements (Dobbins and Feldman, 1994).

## Anatomical and functional mapping in humans

Thanks to recent developments in neuroimaging techniques, direct studies on the brain of living humans, with or without pathology, have become possible using minimally invasive tools. These techniques can be used to study the structure of the brain, e.g., Magnetic Resonance Imaging (MRI) and Computed Axial Tomography, or its function, e.g., Functional Magnetic Resonance Imaging (fMRI), Single Photon Emission Computed Tomography, Positron Emission Tomography, Magnetoencephalography, Electroencephalography and Long Latency Evoked Potentials (Aine, 1995).

Functional neuroimaging studies provide a dynamic view of brain activity. These studies expose the subject to a given stimulus or context, like a cognitive task, to observe its behavior and record the underlying brain activity. Among all, fMRI (Ogawa et al., 1992) has become the most widely used functional neuroimaging technique. fMRI not only allowed to quantify task-dependent activations of brain regions by detecting changes in blood flow, but it also boosted the study of brain networks and connectome. In particular, resting-state fMRI allows the identification of functional connectivity networks between specific areas (Fox and Raichle, 2007; Van Dijk et al., 2010). fMRI is also applicable to non-human species (Gorges et al., 2017) and its use is rapidly extending to rodent research [see for example (Pan et al., 2015, 2018; Grandjean et al., 2020; Pradier et al., 2021)]. Nonetheless, fMRI has limitations and its results are sometimes misinterpreted (Maestú et al., 2003; Logothetis, 2008; Elliott et al., 2020).

Since emotion involves the integration of cognitive, somatomotor, and autonomic functions, we will exploit the privileged point of view offered by human investigations to address the concept of segregation and distribution of these same functions across regions of the cerebellum.

### Neuroimaging studies in human cerebellum

**FIGURE 7:**
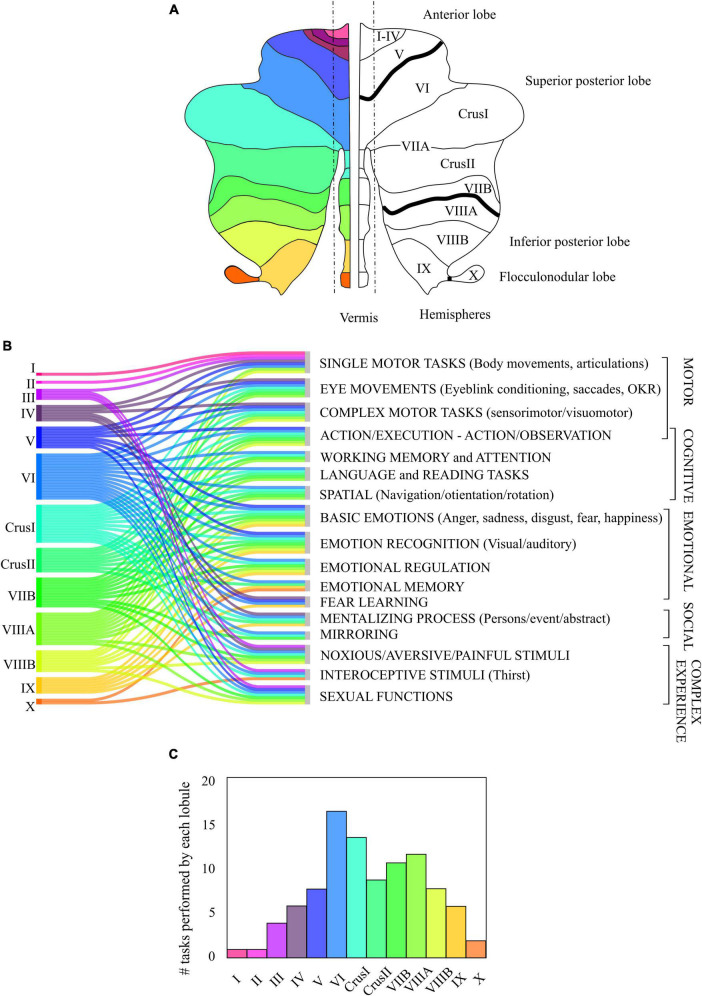
Contribution of the human cerebellum to motor, cognitive, emotional, social and complex task. **(A)** Classical nomenclature of the division of the human cerebellum, from anterior lobe (I to V) to posterior lobe (Superior posterior: lobules VI to VIIB and Inferior posterior: lobules VIIIA to IX) separated by intrabiventer fissures (dark black line), and flocculo-nodular lobe. **(B)** Association of the activation of the different cerebellar lobes (in colors) to different functional tasks, based on the consulted bibliography. The tasks were divided into motor, cognitive, emotional, and social. Complex experiences cannot be strictly classified into motor, cognitive, emotional, or social tasks. **(C)** Lobules involvement in functional tasks of panel **(B)**. The height of each bar corresponds to the number of tasks (out of the 17 listed) related to the activation of each lobule.

A growing literature on functional neuroimaging (Stoodley and Schmahmann, 2009; Guell and Schmahmann, 2020) evaluates structure-function relationships in motor and non-motor areas of the human cerebellum, together with clinical findings. These studies highlight the involvement of different circuits depending on the task being performed. The imaging studies in humans were used to reconstruct a functional topographic map of the cerebellum. The results of these studies can be interpreted according to Larsell’s nomenclature (Schmahmann et al., 1999), that divides the cerebellum along the anterior-posterior axis in lobules denoted by roman numerals from I to X (optional labels, H and v, are used to denote the hemispheres and the vermal region). In addition, the two largest hemispheric lobules are separated in two parts, namely lobule VII (into VIIA comprising crus I and crus II, and VIIB), and lobule VIII (into VIIIA and VIIIB), according to the intrabiventer fissures (Figure 7A). It should be noted that, in human studies, the gross subdivisions of the cerebellum are anterior and posterior, this latter includes superior and inferior parts and the flocculo-nodular lobe.

In general, in functional studies, the cerebellum is usually divided into two representations, motor and non-motor (Stoodley and Schmahmann, 2009; Buckner et al., 2011; Stoodley et al., 2012; Guell et al., 2018b,^2019^; Guell and Schmahmann, 2020). Specifically, areas involved in motor representations are the anterior lobe and parts of lobule VI and VIII, while the areas involved in non-motor representations are lobules VI, crus I, crus II, VIIB and IX with extensions to lobule X. Some authors, such as King et al. (2019), promotes a functional parcellation to re-define lobar boundaries as it better matches the experimental data. Also, Boillat et al., 2020 highlighted that the activation patterns observed in functional studies do not strictly adhere to lobar boundaries, and suggested that the cerebellar cortex is a continuous sheet.

Although extensive fMRI studies have been performed, a composite unified picture of the cerebellar functional topography is still lacking. Figure 7B represents the activation of the different cerebellar lobules in motor, cognitive, emotional, and social tasks. The most significant observation coming from our data metanalysis is that, besides a preference for specific modalities, there is a considerable overlap of functions in several cerebellar areas.

### Motor cerebellum

As commonly used in literature, we distinguish motor tasks into *simple motor tasks*, *eye movements tasks*, and *complex motor tasks*.

Studies on *simple motor tasks* (Riecker et al., 2005; Stoodley and Schmahmann, 2009; Guell et al., 2018a; Boillat et al., 2020) report activation of the anterior lobe and lobule VIII, confirming the electrophysiological studies of body maps (Snider and Eldred, 1951). More specifically:

1. *Right/left leg and foot movements* activate ipsilateral lobules II, III, and IV with extensions to lobule I,
2. *Orofacial movements* activate medial regions of lobule VI,
3. *Right/left hand movements* activate ipsilateral lobules V, VIII,
4. *Tongue movement* activates ipsilateral lobule VI,
5. *Articulation* activates medial hemispheric lobules IV to VI and VIII.

Studies on *eye movements* (Dieterich et al., 2000; Dimitrova et al., 2002; Alvarez et al., 2010; King et al., 2019; Boillat et al., 2020) report activation of the anterior lobe and lobule VIII, including vermal areas and lobule VII. For example: *optokinetic reflex, smooth pursuit, vergence and saccades* activate vermal lobules VI and VII and extend into vermis lobules IV-V and adjacent medial regions of crus I and crus II.

Studies on *complex motor tasks* (Debas et al., 2010; Ma et al., 2010; Schlerf et al., 2010; Stoodley et al., 2012; Hardwick et al., 2013; Ashida et al., 2019; King et al., 2019; Tzvi et al., 2020) report activation of the anterior lobe, lobule VIII as well as lobule VII. More specifically:

1. *Motor learning* activates bilateral anterior/medial cerebellum extending into lobule VI,
2. *Motor planning* activates lobules VI, crus I and crus II,
3. *Motor sequencing* activates anterior regions extending into lobule VI, VIIIA/B and crus II,
4. *Motor adaptation* activates anterior lobules IV-V, VIII bilaterally,
5. *Visuomotor adaptation* activates lobules IV-V, VI, VIII, crus II,
6. *Finger tapping* activates ipsilateral anterior lobe (lobules IV-V) and lobules VI, VIII,
7. *Left and right button press* activates ipsilateral anterior lobe and lobule VIII,
8. *Skilled movement* activates lobules VI, crus I,
9. *Execution* (reported in both motor and cognitive studies) activates lobes V, VI, VIII,

Clinical studies (Urban et al., 2003; Kumral et al., 2007; Schmahmann et al., 2009; Stoodley and Schmahmann, 2009; Maderwald et al., 2012; Bultmann et al., 2014; Stoodley et al., 2016) support the previously reported experimental findings on motor tasks. Affections of these lobules or regions have been additionally found to be related to neuromotor disorders. For example, patients with poor finger tapping have lesions in the ipsilateral anterior cerebellum, ataxia scores correlate with damage of the anterior lobe (lobules II to V extending into lobule VI), and lesions responsible for cerebellar dysarthria include lobules I, II, III.

### Cognitive cerebellum

fMRI analyses in cognitive tasks (Fink et al., 2000; Stoodley and Schmahmann, 2009; Stoodley et al., 2012; Balsters et al., 2014; E et al., 2014; Guell et al., 2018a; Ashida et al., 2019; Casiraghi et al., 2019; King et al., 2019) report vermal activation of lobules VI and VII as well as hemispheric activation of these lobules with lobule VIIIA and extensions to lobule V. More specifically:

1. *Action/execution tasks* (reported in both motor and cognitive studies) activate lobules V, VI, VIII and regions of crus I
2. *Action/observation tasks* activate areas of lobule VI, crus I and crus II,
3. *Working memory* activates hemispheric lobules VI, crus I, and VIIB to VIIIA,
4. *Visuospatial working memory and attention* activate hemispheric lobules VI, crus I,
5. *Linguistic and reading tasks* activate right lobules VI, VII, and VIIIA,
6. *Spatial (mental rotation)* activate left-lateralized lobules VI, VII to VIIIA, crus I and midline VIIA,
7. *Spatial (navigation/orientation/mutual rotation)* activate right crus I, bilateral lobules VI, and VII.

Clinic evidence (Schmahmann and Sherman, 1998; Mariën et al., 2014; Stoodley et al., 2016; Hoche et al., 2018) proves that cognitive impairments correlate with posterior lobe lesions (lobules VI and VII, including crus I, crus II) in abstract reasoning, working memory, planning, executive functioning, spatial cognition, language, and cognitive regulation. Damage to the left hemisphere is more related to spatial difficulties, while the right cerebellar hemisphere to language problems.

### Emotional cerebellum

Most studies (Kilts et al., 2003; Wildgruber et al., 2005; Stoodley and Schmahmann, 2009; Stoodley et al., 2010, 2012; Moulton et al., 2011; Baumann and Mattingley, 2012; Schraa-Tam et al., 2012; Adamaszek et al., 2014, 2017; Nummenmaa et al., 2014; Lange et al., 2015; An et al., 2018; Ernst et al., 2019; King et al., 2019; Thomasson et al., 2019) agree that the regions involved in emotional processing are in the posterior lobes, including the vermal area.

Vermal and paravermal regions are involved in fundamental or primary emotions, namely:

1. *Anger:* vermal lobule IX and paravermal lobules VI, crus I and crus II,
2. *Sadness:* vermal lobules, crus I, crus II and paravermal lobule VI,
3. *Disgust;* vermal lobules V, VIIIA to IX and paravermal lobule VI,
4. *Fear:* vermal lobule VIIB and paravermal lobes VI, crus I and crus II,
5. *Happiness:* paravermal lobe VIIIB.

In the domain of *Emotional recognition:*

1. *Seeing emotional vs neutral images* activates posterior lobes, including lobules VI, VII
2. *Seeing pleasant images* activates the right lobules VI and crus I
3. *Seeing unpleasant images* activates bilateral lobules VI to VIIIB, vermis of lobule IX, and left crus I
4. *Processing angry facial expression* activates the right lobule V
5. *Identifying emotional intonations* activates midline lobule VII and lateral lobules VI and crus I
6. *Listening to other’s spoken emotional narratives* induces activation in lobules VI and IX, right lobules VIIB, VIII, and IX
7. P*rocessing of emotion-laden visual and auditory art* activates crus I and crus II is reported.

*Emotion regulation* activates vermal and hemispheric lobules VI, crus I, VIIB, VIIIA/B and IX. *Emotional memory* activates crus II and lobules VI, IX, X.

Finally, *Fear learning* activates the culmen, right and left hemispheric lobule IV-V and left lobule VI, and right and left lobule hemispheric lobule IX.

### Social cerebellum

The study of the role of cerebellum in social behaviors (Van Overwalle et al., 2014, 2020; Leggio and Olivito, 2018) has recently gained attention. More precisely, on *mentalizing* task which evaluate the ability to understand the mental state – of oneself or others – that underlies overt behavior:

1. *social mentalizing* activates crus I, crus II, and lobule IX,
2. *person mentalizing* activates crus I, lobules IV and VI,
3. *event mentalizing* activates crus I,
4. *abstract mentalizing* activates crus I, lobules VI and IX.

Mirroring is the behavior in which one person subconsciously imitates the gesture, speech pattern, or attitude of another). This task activates crus I, lobules VIIB and VI.

Clinical data (Levisohn et al., 2000; Schmahmann et al., 2007; Tavano et al., 2007; Adamaszek et al., 2014) report the relationship between damage to the posterior vermis with extensions in the medial cerebellar regions with social, behavioral, affective deficits and emotional disorders in patients with affect dulling, anxiety, depression, impulsivity, irritability, aggression and autism spectrum disorder.

### Complex experiences involving multiple cerebellar areas

fMRI studies indicate that the cerebellum responds to other complex experiences such as aversive and painful stimuli (Becerra et al., 1999; Ploghaus et al., 1999; Dimitrova et al., 2003; Singer et al., 2004; Jackson et al., 2005; Moulton et al., 2011; Borsook et al., 2018), interoceptive stimuli (Parsons et al., 2000) as well as sexual experiences (Holstege and Huynh, 2011; Wise et al., 2017).

*Aversive stimuli* (noxious heat, electricity, unpleasant images) activate lobule VI, crus I, VIIB, while *painful stimulation* evokes bilateral activation of lobules III, IV and VIII as well as the vermal and hemispheric parts of lobule VI and crus I. Therefore, pain experiences activate multiple areas: the anterior regions in relation to the motor component as reflex, the posterior regions in relation to the anticipation of pain or unpleasant situation, and the posterior vermis for the painful experience itself including fear or startle reactions. *Pain empathy* activates bilaterally lobule VI and crus I.

*Interoceptive stimuli* such as *thirst* activates vermal lobe III, whereas during *maximal thirst* lobes X, VI and crus I are activated, and the left lobe VI is strongly activated after *drinking to satiety*.

*Man ejaculation* evokes activation in lobule VIII, right vermal lobule V, left vermal lobule VII, left lobule VI, and bilateral crus I, moreover *women orgasm* elicited activity left lobule V and right lobule III, while during the *post-orgasm recovery* activates vermal and hemispheric lobule VI and lobule VII.

**FIGURE 8:**
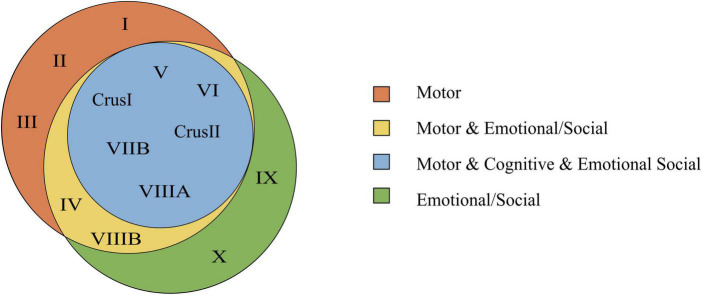
Venn’s diagram based on the consulted bibliography of three functional domains (motor, cognitive, emotional/social). In this diagram, the emotional and social tasks are joined within the same functional domain, and complex experiences are excluded. The Venn’s diagram shows the lobules involved in motor, cognitive, and emotional/social tasks. Overlapping parts contain the lobules that are common to two or all regions. Notice that lobules involved in the cognitive function are in common with both the motor and emotional/social function.

It would be wrong to limit an area of the cerebellum strictly to either motor, cognitive, emotional or social tasks. However, lobules task-dependent predominance can be delineated (Figures 7, 8).

### Lobule VI as an integrating region

Figure 7B shows how the lobule VI is involved in all the reported tasks of our collected literature studies. This lobule might represent an integrating or modulating node between motor, cognitive, emotional, and social areas. This may be due to the connection of this area with multiple cortical and brain stem areas. It would be interesting to see whether a multimodal input implies cells with larger dendrites to integrate more synaptic information.

### Extra cerebellar connectivity

Studies on resting-state functional connectivity showed that most of the cerebellar regions partake in 5 intrinsically-connected circuits or networks (Habas et al., 2009; Brissenden et al., 2018):

1. the *sensorimotor network* (lobule V-VI and VIII),
2. the *default-mode network* (lobules VIIa and IX),
3. the *right and left central executive network* (lobule VIIa),
4. the *dorsal attentional network* (lobules VIIB and VIIIA)
5. the *salience network* (lobules VI and VII).

Furthermore, at least 2 functional macroscale circuits involving the cerebellum have been identified (Leggio and Olivito, 2018): a *sensorimotor zone* (anterior cerebellum) functionally correlated with premotor, motor, somatosensory, visual, and auditory cortical regions, and a *supramodal zone* (posterior cerebellum) functionally correlated with dorsolateral prefrontal and inferior posterior-parietal regions.

Resting-state fMRI studies report connections of the cerebellum with cerebral cortex (Krienen and Buckner, 2009; O’Reilly et al., 2010), striatum (Stoodley and Schmahmann, 2009), basal ganglia (Bostan et al., 2010, 2013), antero-medial and mediodorsal thalamus, and habenula (Milardi et al., 2016; Habas and Manto, 2018). Moreover, tracts have been identified between the medial part of posterior lobule of the cerebellum with insula (Cauda et al., 2011; Baur et al., 2013), frontal operculum, anterior cingulate cortex, medial prefrontal cortex, amygdala, and hippocampus, as well as between the lateral part of the cerebellum with the hypothalamus and anterior cingulate cortex (Sang et al., 2012; Bostan et al., 2013; Murty et al., 2014; Habas, 2018).

### Limitations of *in vivo* neuroimaging studies of the cerebellum

Neuroimaging studies are affected by limited spatial and temporal resolution, although this has recently been increased by technological improvements such as fMRI, electroencephalography (Huster et al., 2012; Jorge et al., 2014; Croce et al., 2016) and advanced analysis methods (Spencer and Goodfellow, 2022). The fine folded pattern of the layers of the cerebellar cortex makes it even more complicated to define cerebellar connectivity in detail in humans. Indeed, the surface of the cerebellar cortex is much more tightly folded than that of the cerebral cortex. Thus, although the visible surface of the cerebellum is several times smaller than that of the neocortex, once unfolded it equals 78% of the total surface area of the neocortex (Sereno et al., 2020). This, together with the extended connectivity to associative cortical areas (Palesi et al., 2015), suggests a prominent role for the cerebellum in the evolution of distinctive human behaviors including cognition and emotion.

## Conclusions and outstanding questions

### Segregation, distribution, and specialization

The evidence reviewed here reveals, as a fundamental aspect of emotional processing in the cerebellum, the interplay of *segregation* and *distribution* of functions in multiple cerebellar regions. The corollary is the concept of *specialization*, that could adapt the general circuit mechanisms of the cerebellum to the specific needs of emotional control.

*Segregation*. Numerous studies (Stoodley et al., 2010, 2021; Solstrand Dahlberg et al., 2020) have shown that lobules of the central cerebellum, in particular lobules VI, VII, and VIII, show most of the fMRI-related activity during emotional tasks. Likewise, the data summarized in Figure 7B indicate that several cerebellar regions are engaged in multiple functions, as opposed to being specific for a single function. It is plausible to ask whether and to what extent the functional specialization of lobules VI, VII, and VIII entails molecular, cellular, or local network specializations as compared to other modules. The search for circuit specializations in the cerebellum may start from central lobules, especially lobule VI that presents the most extensive implications for emotional processing (Figure 7C).

*Distribution*. Since the emotional response entails sensorimotor, cognitive, and autonomic aspects, numerous cerebellar lobules that generically operate motor or autonomic control are indeed also contributing to emotion. As a corollary, some lobules should be convergence sites for emotional, cognitive and motor responses, as we have pointed out for lobule VI.

*Specialization.* The processing role of the cerebellum at system level notwithstanding, the cerebellar network repeats itself in the general motive but not in the details, suggesting that some properties may differentiate microcircuit computation. Focusing on central lobules, the following pattern emerges:

1. Lobules VI and VII are almost entirely Z + (or upbound), PCs have lower firing rate and show PF-PC long-term potentiation. Lobule VIII has alternating Z + /Z- stripes.
2. Lobules VI to X show an increasing amount of PC axonal collaterals contacting GrCs, UBCs, and GoCs.
3. Lobules VI to VIII have almost no UBCs.
4. Lobule VI to VIII have higher cortical thickness than other lobules, and lobule VI has a higher PC density than most of the other lobules.
5. There is an antero-posterior gradient in GrC and PC firing adaptation.

It is possible that these different properties subtend the higher need for multimodal integration of lobules VI-VIII bringing about larger input-output fiber sets, different management of recurrent loops and local processing of signals on specific frequency bands, differential processing of burst and tonic firing depending on the nature of the input, different needs for plasticity generation and memory storage. To what extent these properties impact on microcircuit function and spatiotemporal dynamics is currently unknown and its understanding will require accurate physiological recordings and model simulations.

### Implications for behavioral processing

Since the cerebellum is involved in learning the association of time-correlated events and it is likely to do so in all its modules (D‘Angelo and Casali, 2013), a core hypothesis is that *the cerebellum orchestrates the time domain for the suppression/initiation of fear responses* as much as it does in other conditioning tasks, e.g., in eye-blink classical conditioning. In other words, the cerebellum should perform in emotional control the same operation it performs in motor control and coordinate the many components of the emotional response including motor and visceral reactions. Lobules VI to VIII are involved and studies in rodents support their functional connection to the amygdala, hypothalamus, and periaqueductal gray. Regions IX and X play a major role in orchestrating the autonomic response. In humans, the pattern of explorable emotions is larger than in rodents, but evidence still supports a *specialization* of lobules VI to VIII. In humans, the high resolution of fMRI techniques during tasks has reinforced the concept of a remarkable *distribution and overlapping* of areas involved in emotional control with those controlling motor, cognitive, and social behaviors. In addition to coordinate the components of the emotional response, the cerebellum may likewise be involved in *comparing the cortical commands with their execution* to predict possible errors and correct them. Again, the cerebellum may play the same coordinating role in emotion as it plays in sensorimotor control.

## Author contributions

CCia, YL, and DO: writing – original draft preparation and visualization. ED’A, LM, CCas, and DR: writing – review and editing. ED’A, LM, and CCas: supervision. ED’A: conceptualization. All authors contributed to the article and approved the submitted version.
